# A rapid method for determining protein diffusion through hydrogels for regenerative medicine applications

**DOI:** 10.1063/1.4999925

**Published:** 2018-06-12

**Authors:** Marian H. Hettiaratchi, Alex Schudel, Tel Rouse, Andrés J. García, Susan N. Thomas, Robert E. Guldberg, Todd C. McDevitt

**Affiliations:** 1 Wallace H. Coulter Department of Biomedical Engineering, Georgia Institute of Technology and Emory University, 313 Ferst Drive NW, Atlanta, Georgia 30332, USA; 2 School of Materials Science and Engineering, Georgia Institute of Technology, 315 Ferst Drive NW, Atlanta, Georgia 30332, USA; 3 Parker H. Petit Institute for Bioengineering and Bioscience, Georgia Institute of Technology, 315 Ferst Drive NW, Atlanta, Georgia 30332, USA; 4 School of Chemical and Biomolecular Engineering, Georgia Institute of Technology, 311 Ferst Drive NW, Atlanta, Georgia 30332, USA; 5 The George W. Woodruff School of Mechanical Engineering, Georgia Institute of Technology, 801 Ferst Drive NW, Atlanta, Georgia 30332, USA; 6 Gladstone Institute of Cardiovascular Disease, 1650 Owens Street, San Francisco, California 94158, USA; 7 Department of Bioengineering and Therapeutic Sciences, University of California San Francisco, 600 16th Street, San Francisco, California 94158, USA

## Abstract

Hydrogels present versatile platforms for the encapsulation and delivery of proteins and cells for regenerative medicine applications. However, differences in hydrogel cross-linking density, polymer weight content, and affinity for proteins all contribute to diverse diffusion rates of proteins through hydrogel networks. Here, we describe a simple method to accurately measure protein diffusion through hydrogels, within a few hours and without the use of large amounts of protein. We tracked the diffusion of several proteins of varying molecular weights along the axial direction of capillary tubes filled with alginate, collagen, or poly(ethylene glycol) hydrogels. The rate of protein diffusion decreased with increasing molecular weight. A computational model of protein diffusion through capillary tubes was also created to predict and verify experimental protein diffusion coefficients. This *in vitro* capillary tube-based method of measuring protein diffusion represents a simple strategy to interrogate protein diffusion through natural and synthetic hydrogels and aid in the design of better biomaterial-based delivery vehicles that can effectively modulate protein release.

## I. INTRODUCTION

Hydrogels, which are highly hydrated polymer networks, possess numerous properties that make them amenable to protein and cell encapsulation and are a particularly promising method for the delivery of therapeutics.^1^ Hydrogel scaffolds can be fabricated from a variety of synthetic and natural polymers and cross-linked via physical and chemical interactions, conferring diverse, tunable properties. Hydrogels used for regenerative medicine applications are typically designed to be injectable, to facilitate minimally invasive administration, degradable, to invite robust cell infiltration and material resorption, and mechanically comparable to surrounding tissues. Such hydrogels can be obtained from natural polymers, such as alginate, collagen, methylcellulose, and hyaluronan, and synthetic polymers, such as poly(ethylene glycol) (PEG) and poly(ethylene oxide) (PEO). Furthermore, many polymers employed in hydrogel design, including alginate, collagen, and sulfated glycosaminoglycans, are naturally charged, which can enable electrostatic interactions with proteins and enhance their encapsulation.^2–4^

Protein diffusion through hydrogels plays a significant role in both protein and cell delivery strategies. Release of proteins from hydrogel delivery vehicles is dictated by hydrogel porosity and degradation kinetics, as well as interactions of the protein and material with the surrounding *in vitro* or *in vivo* environment.^5,6^ Effective cell transplantation using hydrogel encapsulation relies on protein diffusion into and out of the hydrogel to ensure cell survival and function. Thus, the evaluation of protein diffusion through hydrogel networks is an important aspect of these delivery systems. Although a wide range of hydrogels with varying properties have been used for the delivery of numerous proteins, information about the diffusivity of therapeutic proteins through specific hydrogel formulations is not always available, often leading to a poor understanding of protein encapsulation and release properties for different materials. Hydrogel formulations and physical properties can differ vastly between research groups, and protein diffusion is usually characterized using model proteins instead of costly therapeutic proteins. Naturally derived polymers often suffer from significant batch-to-batch variability, which can affect their properties, while chemical modifications to polymers such as irradiation, lyophilization, and the addition of functional groups may also impact protein diffusion.^7–11^ Considering these numerous challenges, the ability to easily measure the diffusion of proteins through various hydrogel delivery systems would provide valuable information about critical material properties that regulate protein delivery.

Many advanced biomaterial delivery systems, with or without specific affinities for proteins, have been developed to achieve sustained protein delivery and cell encapsulation.^12–16^ Although understanding the diffusion of an encapsulated protein through a hydrogel delivery system is crucial to effective protein delivery, current methods to evaluate protein diffusion through hydrogels have been limited. The most common method involves long term protein release studies, in which bulk hydrogels containing encapsulated proteins are fabricated, immersed in a solution, and the solution is sampled for protein release over a period of several days to weeks. These data can be fit to established diffusion models to obtain effective diffusion coefficients of the protein from the hydrogel.^17^ Protein diffusion through hydrogels can also be evaluated by measuring the bulk properties of the material such as its refractive index or nuclear magnetic resonance (NMR) spectra.^18,19^ However, these assays are typically only conducted using inexpensive “model” proteins, such as bovine serum albumin (BSA), chymotrypsin, and lysozyme, instead of costly therapeutic proteins, due to the high concentrations of protein (>1 mg/ml) that must typically be used.^20^ Evaluation of a material's diffusivity over long periods of time can be further confounded by protein and material degradation; thus, diffusion coefficients obtained using these methods may not accurately capture protein diffusion through hydrogel networks alone and cannot easily be separated from the effects of material degradation on protein release. Alternatively, fluorescence recovery after photobleaching (FRAP) can be used to evaluate diffusion of fluorescently labeled protein into a region containing protein that has undergone photo-bleaching.^20,21^ Compared to evaluating protein release from bulk hydrogels, FRAP allows greater control over protein diffusion within a smaller area (several microns) and shorter time period (several minutes). However, FRAP is inherently a low throughput method that can only be conducted on a single sample at a time.

In order to gain a better understanding of the kinetics of protein release from hydrogels, a simple method to rapidly determine protein diffusion through various hydrogels was developed. Effective diffusion coefficients were calculated by tracking the axial diffusion of fluorescently labeled proteins through a hydrogel-filled capillary tube over several hours. This method was tested using proteins of various molecular weights, which served as models for a wide range of protein-based therapeutics that could be released from hydrogels. We compared three commonly used hydrogels for protein and cell delivery: alginate,^12^ collagen,^22–24^ and maleimide cross-linked poly(ethylene glycol) (PEG-MAL).^13,15^ The experimental data were used to create a COMSOL model of protein diffusion through capillary tubes and predict the diffusion coefficient of a therapeutic protein of interest, bone morphogenetic protein-2 (BMP-2), through the hydrogels. BMP-2 diffusion coefficients were confirmed using the capillary tube diffusion method. Overall, these results demonstrate a robust and simple method to measure protein diffusion through hydrogels, which can be used to predict the diffusion coefficients of therapeutic proteins. This method could replace larger-scale diffusion experiments, which are typically conducted in conical tubes and well plates and often require large amounts of costly therapeutic proteins and low throughput FRAP-based diffusion experiments. Protein release from biomaterial delivery vehicles could be improved by providing diffusion coefficients specific to the hydrogels being used that are more accurate than those determined from theoretical calculations alone and easier to obtain than using alternative experimental methods. Furthermore, high throughput methods of evaluating protein diffusion are valuable, as they may facilitate optimization of hydrogel formulations that can provide tailored protein release profiles.

## II. RESULTS

### A. Determination of diffusion coefficients of model proteins

**FIG. 1.**
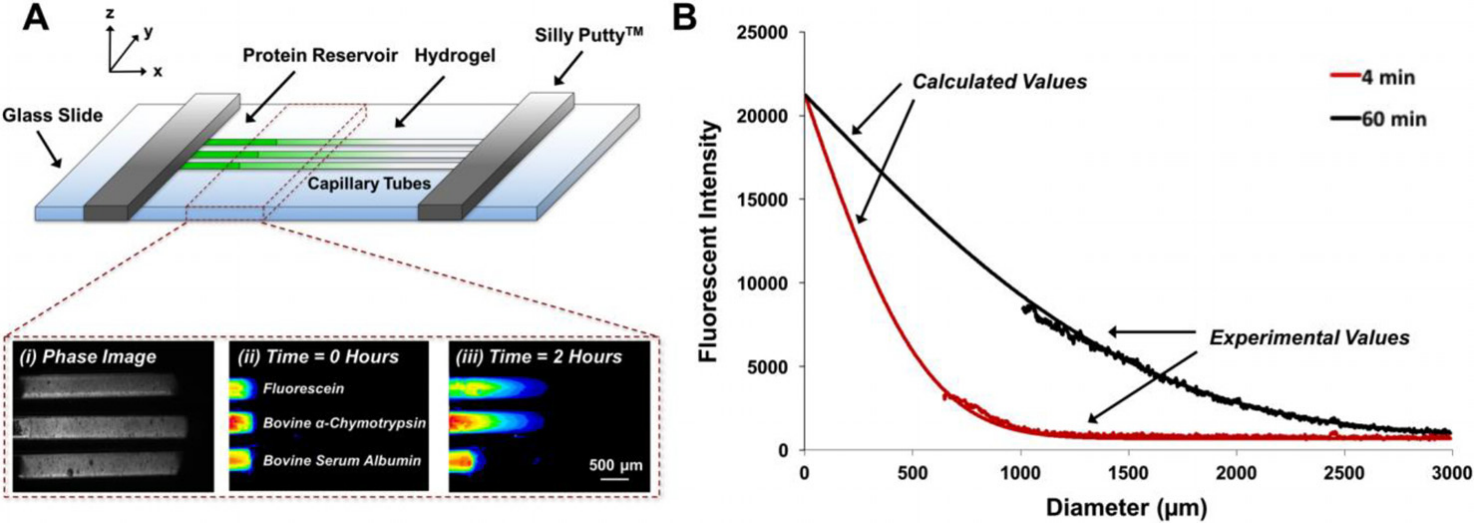
Capillary tube diffusion experimental set-up. (a) For microscope imaging, three capillary tubes filled with hydrogel and protein solution were affixed to a glass slide (approximately 0.5 mm apart) using Silly Putty™. (i) Bright field image of capillary tubes filled with 2% (w/v) collagen hydrogels. Fluorescence [green fluorescent protein (GFP) channel] images of free fluorescein dye, fluorescein-labeled bovine α-chymotrypsin, and fluorescein-labeled bovine serum albumin diffusing through collagen hydrogels at (ii) 0 h and (iii) 2 h. (b) Experimental profiles of fluorescence intensity signals obtained from images taken 4 and 60 min after the start of protein diffusion. Fluorescence intensity signals were normalized to the signal of the protein reservoir. Theoretical profiles of normalized fluorescence calculated using a one-dimensional model of diffusion [Eq. (1)] for diffusion through capillary tubes. Experimental and calculated profiles are comparable. (Chi-square tests, p = 0.95 at 4 min, p = 0.99 at 60 min.)

Effective diffusion coefficients of proteins through 2% alginate, 6% collagen, and 4% PEG-MAL hydrogels were calculated for fluorescein (0.3 kDa^25^) BMP-2 (26 kDa^26^) and several model proteins of varying molecular weights [bovine α-chymotrypsin (αCT)—25 kDa,^27^ bovine serum albumin (BSA)—66 kDa,^28^ and human immunoglobulin G (IgG)—150 kDa^29^] by fitting the time- and space-dependent changes in fluorescence intensity throughout the hydrogel to a one-dimensional model of diffusion [Eq. (1)].^30,31^ Representative images of fluorescein, αCT, and BSA diffusing through collagen hydrogels at 0 and 2 h reflect the relatively fast diffusion of fluorescein, moderate diffusion of αCT, and slow diffusion of BSA [Fig. 1(a)]. Sample normalized fluorescence intensity curves obtained from experimental data and the one-dimensional model of diffusion [Fig. 1(b)] are comparable at low fluorescence intensities, farther away from the protein-hydrogel interface (p = 0.95 at 4 min, p = 0.99 at 60 min), and demonstrate the development of an expected length-dependent fluorescence intensity profile in the capillary tube over time.

**FIG. 2.**
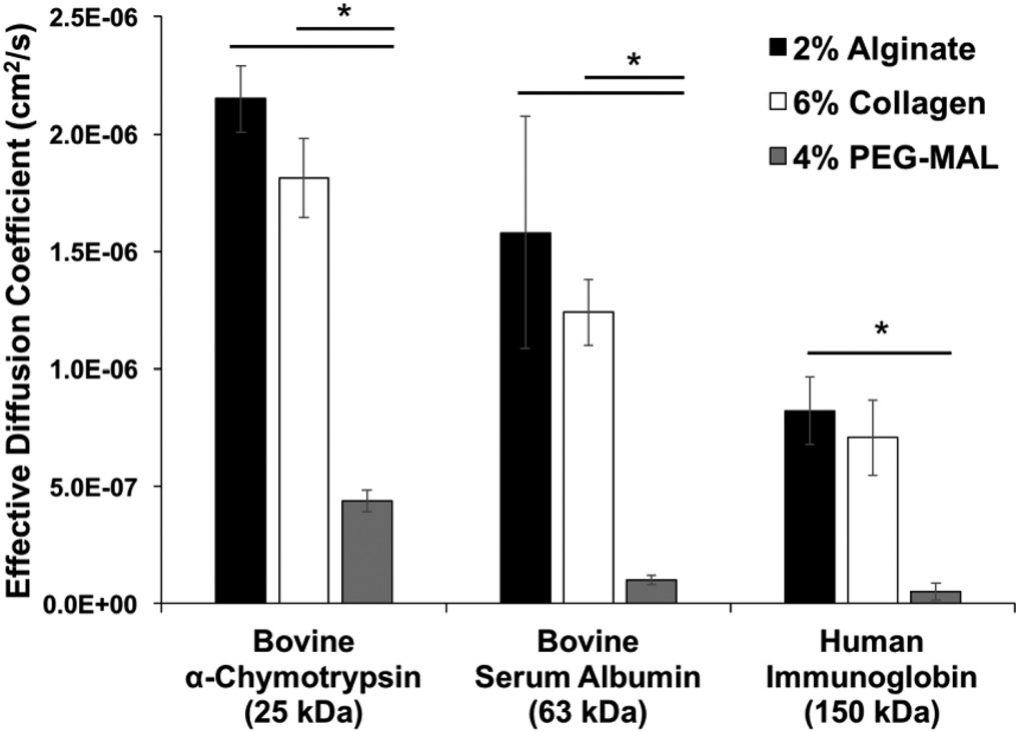
Effective diffusion coefficients of model proteins in hydrogels. Effective diffusion coefficients of fluorescein-labeled model proteins (bovine α-chymotrypsin, bovine serum albumin, and human immunoglobulin G) in 2% (w/v) alginate, 6% (w/v) collagen, and 4% (w/v) PEG-MAL hydrogels. Diffusion coefficients were determined using image analysis of fluorescently labeled biomolecules diffusing through capillary tubes filled with hydrogels, followed by curve fitting to a one-dimensional model of diffusion [Eq. (1)]. (* = p < 0.05 as indicated; n = 3.)

All model proteins exhibited significantly faster diffusion in collagen and alginate hydrogels compared to PEG-MAL hydrogels (Fig. 2). Fluorescein displayed a higher diffusion coefficient than all model proteins diffusing through either alginate or collagen hydrogels (Fig. S3) but not through PEG-MAL hydrogels. Overall, the type of hydrogel had a significant effect on protein diffusivity, and effective diffusion coefficients decreased with increasing protein molecular weight, as expected and previously demonstrated.^32–34^

### B. COMSOL model of protein diffusion in capillary tubes

**FIG. 3.**
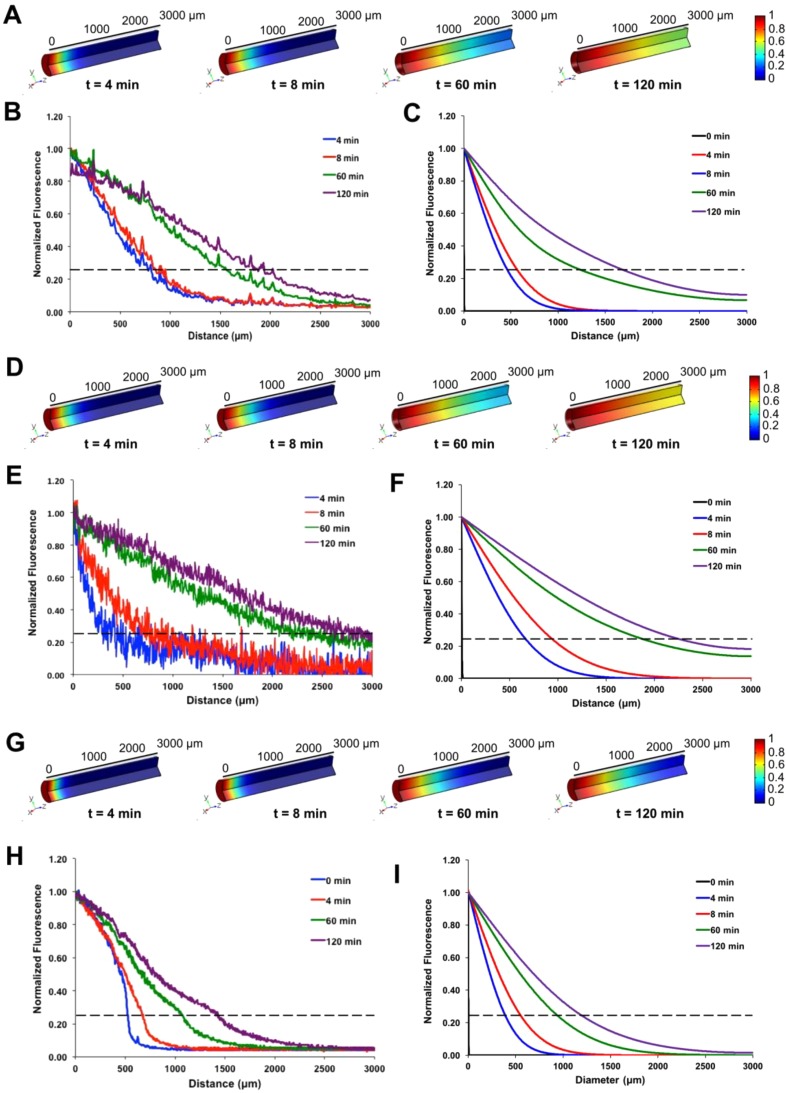
Comparison of experimental and computational diffusion profiles of α-chymotrypsin through hydrogels. COMSOL models of capillary tubes containing (a) 2% alginate, (d) 6% collagen, and (g) 4% PEG-MAL were generated over 2 h and used to evaluate theoretical diffusion of α-chymotrypsin using the effective diffusion coefficients determined experimentally. Experimental fluorescence profiles [(b), (e), and (h)] were found to not differ significantly compared to COMSOL generated protein profiles [(c), (f), and (i)]. Dashed lines indicate 25% fluorescence threshold below which experimental data were used to calculate diffusion coefficients. (Chi-square tests, p = 0.98 for 2% alginate, p = 0.95 for 6% collagen, and p = 0.72 for 4% PEG-MAL).

A mathematical model describing protein diffusion through hydrogels in capillary tubes was created to determine whether the diffusion coefficients obtained from experimental data using Eq. (1) could accurately describe the axial progression of fluorescence intensity profiles in the capillary tubes over time. Fluorescence intensity profiles for fluorescently labeled αCT were obtained experimentally and used to calculate diffusion coefficients for αCT through 2% alginate, 6% collagen, and 4% PEG-MAL hydrogels. These diffusion coefficients were then used in COMSOL models to generate theoretical fluorescence intensity profiles for αCT through the hydrogels. Experimental fluorescence intensity profiles (left graphs) matched theoretical fluorescence intensity profiles (right graphs) for αCT diffusion through 2% alginate [Figs. 3(a)–3(c); p = 0.98], 6% collagen [Figs. 3(d)–3(f); p = 0.95], and 4% PEG-MAL [Figs. 3(g)–3(i); p = 0.72] hydrogels. COMSOL model results closely matched theoretical curves calculated from Eq. (1) for all hydrogels (p = 0.98–1). Both experimental and theoretical data, as well as three-dimensional COMSOL representations of hydrogel-filled capillary tubes, revealed faster development of fluorescence through alginate and collagen hydrogels compared to PEG-MAL hydrogels (Fig. 3), as expected based on the higher diffusion coefficients obtained for αCT diffusion through these hydrogels (Fig. 2).

### C. Determination of diffusion coefficients of BMP-2

**FIG. 4.**
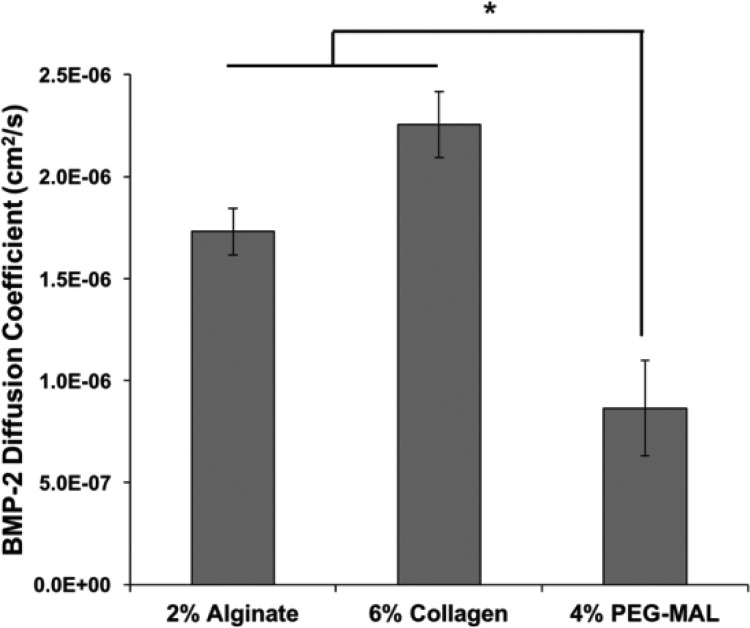
Effective diffusion coefficients of BMP-2 through hydrogels. Effective diffusion coefficients of BMP-2 diffusing through 2% (w/v) alginate, 6% (w/v) collagen, and 4% (w/v) PEG-MAL hydrogels. Diffusion coefficients were determined using image analysis of fluorescently labeled BMP-2 diffusing through capillary tubes filled with hydrogel, followed by curve fitting to a one-dimensional model of diffusion [Eq. (1)]. (* = p < 0.05 as indicated; n = 3.)

Effective diffusion coefficients of a therapeutic protein of interest, BMP-2, were predicted for each hydrogel using the diffusion coefficient of αCT, which has a similar molecular weight and isoelectric point to BMP-2 (αCT: MW = 25 kDa, pI = 8.75;^35^ BMP-2: MW = 26 kDa, pI = 9.15^36^). Experimentally determined BMP-2 diffusion coefficients were not statistically different from experimentally obtained diffusion coefficients of αCT in 2% alginate (p = 0.69), 6% collagen (p = 0.72), and 4% PEG-MAL (p = 0.32) hydrogels. Similar to αCT, diffusion of BMP-2 through PEG-MAL hydrogels was significantly slower than that through alginate and collagen hydrogels (Fig. 3); the slower BMP-2 diffusion may be attributed to a smaller mesh size and structural differences caused by the branched nature of the smaller PEG chains (∼5 kDa) compared to the larger, more fibrillar collagen and alginate monomers (50–200 kDa).^7,37,38^ As expected, correlations between the protein molecular weight and diffusion coefficients led to more accurate diffusion coefficients for BMP-2 in 2% alginate and 6% collagen hydrogels (1.73 × 10^−6^ cm^2^/s compared to 2.15 × 10^−6^ cm^2^/s and 2.25 × 10^−6^ cm^2^/s compared to 1.81 × 10^−6^ cm^2^/s, respectively) than in 4% PEG-MAL hydrogels (8.65 × 10^−7^ cm^2^/s compared to 4.38 × 10^−7^ cm^2^/s) (Fig. 4).

## III. DISCUSSION

Tissue engineering strategies that involve delivery of bioactive proteins and cells rely heavily on the ability to design biomaterial delivery vehicles that can provide controlled protein release and retention. Many hydrogel delivery systems are largely or solely diffusion-controlled, meaning that the release rate of the encapsulated protein is dictated by the ability of the protein to traverse the pores of the polymer network.^39^ The effect of the protein release rate on biological outcomes has been previously demonstrated in numerous systems, including BMP-2 delivery for bone regeneration,^12^ vascular endothelial growth factor (VEGF) delivery for therapeutic vascularization,^40^ and nerve growth factor (NGF) delivery for neurite extension.^41^ Thus, understanding the diffusivity of therapeutic growth factors through hydrogel networks is an important consideration in developing delivery systems for regenerative medicine applications. A simple and robust method for determining protein diffusion through hydrogels was established to fulfill the need to calculate effective diffusion coefficients for therapeutic proteins through common biomaterial delivery vehicles. Since alginate and collagen are naturally derived materials that exhibit high intrinsic and post-processing variability^8,42,43^ and PEG hydrogels can exhibit variable properties depending on cross-linking chemistry and extent, the ability to experimentally determine diffusion coefficients for the specific polymers used in our studies could provide more accurate information than theoretical diffusion coefficients often obtained from the literature.^32^

The equation describing one-dimensional diffusion that was chosen for our analysis has typically been applied to diffusion of proteins through *in vivo* capillary beds and has been widely used to investigate interstitial transport in normal and neoplastic tissues.^31^ Previously, diffusion of gelatin nanoparticles through collagen hydrogels in capillary tubes has been investigated as an *in vitro* model of nanoparticle penetration through collagen-rich tumor tissues.^30^ We sought to adapt this *in vitro* model of tumor transport so that it could be more broadly applied as a simple method of investigating protein diffusion through various hydrogels. The capillary tube diffusion method was successfully used to evaluate the effective diffusion coefficients of several model proteins in addition to a therapeutic protein of interest (BMP-2) in alginate, collagen, and PEG-MAL hydrogels since these hydrogels have previously been used as BMP-2 delivery vehicles.^12,15,44^

When the experimentally determined diffusion coefficients for α-chymotrypsin through the hydrogels were input to a COMSOL model of the capillary tube set-up, the theoretical fluorescence profiles were found to be comparable to experimental profiles, demonstrating that similar data could be generated using mass transport principles. When these diffusion coefficients were compared with diffusion coefficients found in the literature for model proteins through free solution and various hydrogels, the values were found to be within the same order of magnitude;^37,45–47^ however, the diffusion coefficients determined using the capillary tube diffusion method were typically found to be higher (1 to 3-fold). Relevant diffusion coefficients from the literature are summarized in Table I. There are many possible technical reasons for these discrepancies. First, the majority of studies that investigated protein diffusion through hydrogels used FRAP or evaluated long term protein release from the hydrogels. In these studies, the protein was encapsulated with the hydrogel and diffusion out of the hydrogel was measured. Entrapment within the hydrogel matrix may slow protein diffusion due to low affinity interactions with the polymer (e.g., van der Waals forces) and steric interference, resulting in non-Fickian diffusion.^46^ However, in our studies, protein from an aqueous reservoir diffused along a single plane into an empty hydrogel, and the COMSOL model reveals that it can be described by Fickian diffusion principles. Protein diffusion through hydrogels is measured under distinctly different conditions from protein diffusion out of hydrogels, with different boundary conditions, geometries, and concentration gradients driving diffusion. In other words, axial diffusion of a protein into a small capillary from a large aqueous reservoir would involve different driving forces than diffusion from the surface of bulk hydrogel into a large aqueous sink; moreover, these scenarios are described by different mass transport equations, which are then used to calculate diffusion coefficients (i.e., one-dimensional diffusion vs. diffusion from an infinite slab).

**TABLE I.** Literature diffusion coefficients of proteins through aqueous solutions and various hydrogels.

Protein	Temperature (°C)	Protein concentration (mg/ml)	Polymer	Polymer concentration (% w/v)	Measurement method	Diffusion coefficient (cm^2^/s × 10^6^)	References
Fluorescein	25	0.0025–0.01	Hydroxyethylcellulose	2.7	Fluorescence intensity analysis	2.6	58
Chymotrypsin	37	0.06	Fibrillar collagen	3.5	Protein release from hydrogel	0.95	49
BSA	20	4	Free solution	N/A	FRAP	0.57 ± 0.02	33
BSA	20	4	Sulfated agarose	6	FRAP	0.27 ± 0.04	33
BSA	37	2	Glycidyl methacrylate-hyaluronic acid, PEG	1–2	Protein release from hydrogel	0.454 ± 0.042 0.085 ± 0.036	17
BSA	37	2	Collagen	1–4.5	FRAP	0.2–0.8	59
BSA	37	2	Free solution	N/A	Protein release from hydrogel	0.914	17
IgG	37	1	Polyvinyl alcohol	3	Protein release from hydrogel	0.1	60

Protein aggregation may present another confounding factor in diffusion experiments. For example, protein aggregation has been shown to be inversely correlated with the extent of protein release from poly(lactic-co-glycolic acid) (PLGA) microspheres.^48^ Others have reported aggregation of model proteins such as BSA at the high concentrations required for protein release experiments (≥1 mg/ml).^17^ Since protein aggregation would result in solutes with a larger hydrodynamic radius, this may also contribute to a slower observed diffusion coefficient. Finally, it is important to note that many other variations in the set-up of diffusion experiments may contribute to larger differences in observed diffusion coefficients, including (1) differences in the properties of the specific polymer batch, polymer weight percentage, and cross-linking method for each hydrogel and (2) differences in the temperature and boundary conditions with which diffusion experiments were conducted. When similar geometry, polymer concentrations, and concentration gradients are considered, protein diffusion may be more comparable. For example, protein release experiments using collagen hydrogels of similar densities demonstrated that diffusion was not significantly hindered by the fibrillar collagen network and diffusion coefficients were comparable to that of water.^37,49^ High guluronic acid alginates, such as the one used in this study, have also been shown to be more porous and result in higher rates of protein diffusion.^38^ These results are similar to what we observed in our own experiments using both collagen and alginate hydrogels, wherein diffusion of model proteins in the hydrogels was relatively fast and similar to diffusion through aqueous solutions.^50^

Ultimately, the utility of this capillary tube-based method of diffusion determination lies in its ease-of-use, cost-effectiveness, and ability to eliminate confounding factors, such as protein and material degradation. Traditional short-term methods of evaluating protein diffusion through biomaterials, such as FRAP and NMR, require complex and expensive set-ups. Alternatively, long term *in vitro* protein release studies are time consuming and can be expensive since they typically require large amounts of protein. The capillary tube-based diffusion method described herein uses fluorescence-based detection, which requires small amounts of fluorescently labeled protein (∼1–2 *μ*g) in a total volume of less than 50 *μ*l (protein reservoir and hydrogel), and can be assessed within a short time frame using a standard fluorescence microscope during which minimal material and protein degradation is expected to occur (2 h). While the capillary tube method may not be able to resolve small differences in protein diffusion through similar hydrogels, this method is valuable for optimizing hydrogel formulations for desired protein release profiles, prior to evaluating protein release from bulk hydrogels on a larger scale. For example, hydrogels with varying physical properties, such as polymer concentration and cross-linking density, can be fabricated in small quantities and tested in a high throughput manner.

Using the capillary tube diffusion method, we found that diffusion of model proteins through hydrogels decreased with increasing molecular weight, as expected and previously observed.^32–34^ No differences were detected between diffusion in 2% (w/v) alginate and 6% (w/v) collagen hydrogels, whereas diffusion of several model proteins, as well as free fluorescein, was significantly slower in 4% (w/v) PEG-MAL hydrogels. Slower protein diffusion in PEG hydrogels may be attributed to a smaller hydrogel mesh size due to the branched nature of the PEG chains. The macromer branch length has been previously shown to impact diffusion through PEG-MAL hydrogels.^51^ The four-arm PEG-MAL used within this study contained uniformly spaced cross-linking sites at the end of each ∼5 kDa branch. On the other hand, individual collagen fibers (∼130 kDa) and alginate chains (∼50 kDa) are more fibrillar in nature.^7,37,38^ Larger, non-uniform meshes are typically formed by these natural materials, which rely heavily on physical entanglement as well as electrostatic interactions for cross-linking, and the spacing between cross-links can vary. These parameters result in a relatively porous, swollen hydrogel network and more variable mass transport properties, which are reflected by the increased standard deviation in diffusion coefficients obtained in alginate and collagen hydrogels compared to PEG-MAL hydrogels.

BMP-2 is often used clinically to stimulate bone regeneration following severe bone loss,^52,53^ and it has been demonstrated that sustained BMP-2 delivery is required for substantial bone regeneration to occur.^12^ Thus, we chose to computationally and experimentally evaluate BMP-2 diffusion through 2% (w/v) alginate, 4% (w/v) PEG-MAL, and 6% (w/v) collagen hydrogels—all of which have been previously used as BMP-2 delivery vehicles.^12,15,22^ The diffusivity of alginate and collagen hydrogels for BMP-2 was similar when evaluated using the capillary tube technique, suggesting that electrostatic interactions between alginate and BMP-2 may be similar to the weak electrostatic interactions displayed between collagen and BMP-2.^52,53^ While our collagen hydrogels were fabricated to be similar in weight percent to typical collagen sponges used clinically (6% w/v),^37,54,55^ there could very likely be differences in the structure of the hydrogel and hydrated sponge which contribute to differences in BMP-2 diffusion. Moreover, in these experiments, alginate and collagen hydrogels were fabricated without protein, and BMP-2 was allowed to diffuse into the matrix; however, in typical BMP-2 delivery systems, the protein is encapsulated within the hydrogel before formation and can interact with moieties that may eventually be cross-linked with calcium ions or through physical entanglement. In contrast, PEG-MAL hydrogels demonstrated significantly slower BMP-2 diffusion than alginate and collagen hydrogels. This corroborates previous results obtained *in vivo*, which demonstrated that BMP-2 retention was increased using a PEG-MAL delivery vehicle compared to a collagen sponge delivery vehicle.^15^

A COMSOL model of protein diffusion through hydrogel-filled capillary tubes was developed to complement experimentally obtained diffusion coefficients based on physical principles; however, in the future, the model could be used to predict protein diffusion through capillary tubes, given the diffusion characteristics of a protein of similar size and charge. Computational modeling of αCT diffusion through alginate, collagen, and PEG-MAL hydrogels based on experimental diffusion coefficients revealed similar development of fluorescence intensity over time between experimental and theoretical profiles. Since αCT and BMP-2 possess similar isoelectric points and sizes, computational results for the model protein were used to further inform and predict the diffusion characteristics of our therapeutic protein of interest, BMP-2. Furthermore, computational modeling provides a simple method of confirming whether the experimental results obtained are realistic since the results obtained from standard protein release assays can be confounded by protein and material degradation. Future studies could aim to combine computational modeling with information about a hydrogel's charge and mesh size to enhance the ability to predict protein diffusion through various networks in lieu of or in addition to conducting diffusion experiments.

## IV. CONCLUSION

The capillary tube diffusion technique developed within this work provides a robust method for determining protein diffusion coefficients through hydrogels over a short period of time and without variability introduced by hydrogel and protein degradation that is typically observed with the use of traditional protein release assays conducted at a larger scale and over longer periods of time. Combined with computational modeling, the capillary tube diffusion technique can predict the diffusion coefficients of therapeutic proteins quickly and cost-effectively, by evaluating small amounts of costly proteins of interest. While other fluorescence-based diffusion analyses often require microfluidic platforms^56^ or confocal microscopes to perform FRAP,^21^ this method can be easily performed using a laboratory microscope with a standard set of objectives and a temperature controlled stage. The simplicity of this method enables rapid characterization of *in vitro* protein diffusion through hydrogel biomaterials and thus aids in the high throughput optimization of biomaterial delivery vehicles to provide tailored protein release.

## V. METHODS

### A. Fluorescent labeling of proteins

Proteins were fluorescently labeled for microscopy using *N*-hydroxy-succinimidyl (NHS)-fluorescein (5/6-carboxyfluorescein succinimidyl ester; Thermo Fisher Scientific; Ex: 494 nm, Em: 518 nm). Human immunoglobulin G (IgG, MW = 150 kDa; Sigma Aldrich), bovine serum albumin (BSA, MW = 66 kDa; Sigma Aldrich), and bovine α-chymotrypsin (αCT, MW = 25 kDa; Sigma Aldrich) were reconstituted at 50 nM (7.5, 3.33, and 1.25 *μ*g/ml, respectively) in 100 mM NaPO_4_ (pH = 8.5), while human recombinant BMP-2 (MW = 25 kDa; R&D Systems) was reconstituted in the same buffer at 10 nM (0.26 *μ*g/ml) due to its reduced solubility at basic pH. NHS-fluorescein was reconstituted in dimethyl sulfoxide (DMSO) at 2 mM and diluted in sodium phosphate buffer to 750 nM for labeling IgG, BSA, and αCT and 150 nM for labeling BMP-2 to achieve 15 times molar excess of label to protein. The reaction proceeded at room temperature in the dark for 2 h in a total reaction volume of 100 *μ*l before the unreacted label was removed via gel filtration through Zeba Spin Desalting Columns with a 7 kDa molecular weight cut-off (Thermo Fisher Scientific). Protein labeling and removal of unreacted dye were confirmed by fractionation through a PD-10 desalting column (GE Healthcare Bio-Sciences), followed by quantification of fluorescence of each molecular weight fraction of the protein solution using a Synergy H4 microplate reader (Biotek) (Fig. S1). Proteins were stored in 100 mM NaPO_4_ at a concentration of 10–50 nM at 4 °C until use.

### B. Preparation of hydrogels

Alginate functionalized with Arginylglycylaspartic acid (RGD) (FMC Biopolymer, Philadelphia, PA), which has previously been used for BMP-2 delivery in a rat femoral bone defect,^12^ was reconstituted at 2% (w/v) in a 5:1 solution of Minimal Essential Media–Alpha Modification (αMEM; Thermo Fisher Scientific) and 4 mM hydrochloric acid (Sigma Aldrich). The average alginate molecular weight was 50 kDa, composed of 65% guluronic acid and 35% mannuronic acid, and was functionalized with 0.016 *μ*mol RGD/mg of polymer. For alginate cross-linking via calcium chloride, hollow borosilicate tubes (L: 100 mm, ID: 600 *μ*m; VitroCom, Mountain Lakes, NJ) were directly immersed in ∼100 *μ*l of non-cross-linked 2% alginate solution such that liquid was drawn up via capillary action to fill ∼3–4 cm of each tube. An insulin syringe with a small needle (3/10 ml, 8 mm length, 0.13 mm inner diameter, BD, Franklin Lakes, NJ) was used to inject a solution of 100 mM calcium chloride into the tubes in direct contact with the alginate solution. Calcium chloride was allowed to diffuse into the alginate solution overnight at 4 °C to promote calcium-mediated cross-linking.

Collagen type I from the rat tail (Corning) in 0.02 N acetic acid at 8%–11% (w/v) was mixed with 1 N sodium hydroxide and 170 mM ethylenediaminetetraacetic acid (EDTA) on ice to obtain a final collagen solution of 6% (w/v) at a neutral pH (∼7.4), as described in the study by Wong *et al.* (2011).^30^ Hollow borosilicate tubes (L: 100 mm; ID: 600 *μ*m) were immersed in ∼100 *μ*l of collagen solution such that liquid was drawn up via capillary action to fill ∼3–4 cm of each tube. Tubes were incubated in a humidified incubator at 37 °C with 5% CO_2_ overnight to promote collagen gelation.

Four-arm PEG-MAL (20 kDa; Laysan Bio, Arab, AL) was reconstituted in 20 mM HEPES (pH = 7.4) at 8% (w/v) and mixed with an equal volume of VPM cross-linker peptide (GCRDVPMSMRGGDRCG; AAPTEC, Louisville, KY) in 20 mM HEPES (pH = 7.4) at 1.2% (w/v) to get a final PEG-MAL solution of 4% (w/v), which has been previously used for BMP-2 and VEGF delivery in a mouse radial bone defect model.^13,15^ Hollow borosilicate tubes (L: 100 mm, ID: 600 *μ*m) were immersed in ∼100 *μ*l of PEG-MAL solution such that liquid was drawn up via capillary action to fill ∼3–4 cm of each tube. The cysteine groups on the VPM peptide reacted with the maleimide groups on the PEG macromer at room temperature at an acidic pH for at least 15 min until gelation had occurred.

### C. Protein diffusion through hydrogels in capillary tubes

Fluorescently labeled proteins were diluted to 50 *μ*g/ml in phosphate buffered saline (PBS; Corning) for capillary tube diffusion experiments. After tubes were filled with alginate, collagen, or PEG-MAL hydrogels, an insulin syringe with a small needle (3/10 ml, 8 mm length, 0.13 mm inner diameter) was used to inject the protein solutions into the tubes in direct contact with the hydrogels. Tubes were tapped gently to remove air bubbles from the interface. Three tubes were then placed on a glass microscope slide approximately 0.5 mm apart with the protein-hydrogel interfaces aligned along the y-axis. The ends of the tubes were affixed to the glass slide and sealed along the open edges using Silly Putty™ (Crayola, Easton, PA) as shown [Fig. 1(a)] to minimize evaporation of the protein solution or water loss from the hydrogels over 2 h. Preparation of the slides was completed quickly to minimize the passage of time between the contact of the protein solution and the hydrogel prior to imaging.

Slides were imaged using a Zeiss AxioObserver XLmulti-S1 inverted microscope (Carl Zeiss, Jena, Germany) with Zeiss MTB2004 64 bit software and an incubated stage. Images were taken at 37 °C using a 2.5x objective lens (numerical aperture: 0.075) and a GFP channel (excitation: 470/40 nm; emission: 525/50 nm). A fluorescence image was taken every 4 min over a period of 2 h to yield a total series of 31 images. Hydrogels were visually inspected under the 2.5× objective lens at the end of the experiment to ensure that no macroscopic structural changes had occurred during the period of imaging. Diffusion of free fluorescein dye (MW = 330 Da; Thermo Fisher Scientific) alone was also evaluated as a small molecule control.

### D. Calculation of effective diffusion coefficients in capillary tubes

Images from the capillary tube diffusion experiments were analyzed using ImageJ Software (NIH, Bethesda, MD). Images were opened as a stack and converted to the gray scale before a line was drawn through the long axis of each tube to define a region of interest for fluorescence intensity measurements. The fluorescence intensity across each tube was measured within the microscope field of view (3 mm) and recorded for all 31 images, yielding a matrix of fluorescence intensity values at each time and distance. Fluorescence intensity throughout the hydrogel was normalized to the average fluorescence intensity displayed in the protein reservoir, which remained constant throughout the duration of the experiment.

Diffusion of proteins through hydrogels in capillary tubes was approximated as one-dimensional diffusion from an infinite protein reservoir since the length of the hydrogels (∼3–4 cm) was much greater than their diameter (∼600 *μ*m),^17,57^ and the fluorescence intensity of the protein reservoirs did not change over the course of the experiments. Thus, diffusion through capillary tubes could be described using the following equation:^30,31^

$$F\left(x,t\right)\propto \text{erfc}\left(\frac{x}{2\sqrt{D_{eff}t}}\right),$$ (1)

in which *erfc* is the complementary error function, F is the fluorescence normalized to the initial time point (dimensionless), x is the distance from the protein reservoir (cm), t is the time since the protein-hydrogel contact (s), and D_eff_ is the effective diffusion coefficient (cm^2^/s). A custom MATLAB code (supplementary material) was used to fit normalized fluorescence intensity values from all 31 measured time points to the complementary error function; specifically, the *fmincon* function was used to perform constrained non-linear curve fitting, evaluate the complementary error function, and determine D_eff_. Previous studies have demonstrated that a non-linear relationship between fluorescence intensity and protein concentration at high concentrations can be a source of error in diffusion analyses.^50^ We observed a similar non-linear relationship between the fluorescence intensity and the concentration of fluorescein-labeled BSA in 6% collagen hydrogels (Fig. S2). A linear relationship was observed at <12.5 *μ*g/ml of protein, which is 25% of the concentration of the protein reservoir used in our capillary tube experiments. Considering this source of error at high protein concentrations near the protein reservoir-hydrogel interface, we limited our analysis to the sections of the fluorescence profiles farther away from the interface (>0.5 mm away) and at lower fluorescence intensity values (<25% of fluorescence in the protein reservoir), as shown in Fig. 1(b). Data that did not meet these criteria were removed prior to MATLAB analysis.

### E. COMSOL diffusion model

COMSOL Multiphysics software (Version 5.1.0.180; Burlington, MA) was used to develop a mathematical model to describe diffusion of proteins through hydrogels in capillary tubes. The three-dimensional geometry of the capillary tube filled with hydrogel (cylinder, D = 600 *μ*m, L = 30 mm) and protein (cylinder, D = 600 *μ*m, L = 10 mm) was reduced to a two-dimensional modeling domain consisting of a singularly divided rectangular domain with axial symmetry along the length of the domain. The Transport of Dilute Species Physics module was used to model diffusion. Since visual inspection did not reveal differences in gel appearance over time, it was assumed that the hydrogel did not appreciably degrade in the time frame investigated (2 h); thus, D_eff_ was not time-dependent. Hydrogels were modeled as liquids in an unmixed batch reactor. It was assumed that the protein concentrations were equal at the interface between the hydrogel and the protein reservoir (C_protein,hydrogel_ = C_protein,resevoir_).

### F. Statistical analysis

All data are reported as mean ± standard error of the mean. Diffusion experiments were run with a minimum of three independent experiments for each group. Statistical significance between diffusion coefficients was determined using one-way or two-way analysis of variance (ANOVA) as appropriate, followed by Bonferroni's post hoc analysis (Graphpad Prism, Version 5.0, La Jolla, CA). Chi-square tests were used to determine whether experimental fluorescence intensity profiles differed significantly from the values calculated using Eq. (1) and COMSOL-derived fluorescence intensity profiles. Chi-square tests were also used to determine whether calculated and COMSOL-derived fluorescence intensity profiles differed from each other. p < 0.05 was considered statistically significant. The *fmincon* function in MATLAB, which was used to fit the diffusion data to Eq. (1), estimated diffusion coefficients based on values that minimized the error (sum of squared residuals) between experimental fluorescence intensities and calculated fluorescence intensities [F(x,t) in Eq. (1)].

### G. Ethics approval

No ethics approval was required for these experiments.

## VI. SUPPLEMENTARY MATERIAL

See supplementary material for supporting information on the purification of fluorescently labeled proteins, relationship between fluorescence intensity and protein concentration, diffusion coefficients of free fluorescein through hydrogels, and MATLAB code used to analyze fluorescence intensity profiles.
